# Traditional healer treatment of HIV persists in the era of ART: a mixed methods study from rural South Africa

**DOI:** 10.1186/s12906-017-1934-6

**Published:** 2017-08-30

**Authors:** Carolyn M. Audet, Sizzy Ngobeni, Ryan G. Wagner

**Affiliations:** 1 Vanderbilt Institute for Global Health, Vanderbilt University, 2525 West End Avenue, Suite 750, Nashville, TN 37203-1738 USA; 2 MRC/Wits Agincourt Research Unit, School of Public Health, Faculty of Health Sciences, University of Witwatersrand, Johannesburg, South Africa

**Keywords:** HIV/aids, Traditional healer, South Africa, Medical pluralism

## Abstract

**Background:**

Human immunodeficiency virus (HIV) substantially contributes to the burden of disease and health care provision in sub-Saharan Africa, where traditional healers play a major role in care, due to both their accessibility and acceptability. In rural, northeastern South Africa, people living with HIV often ping-pong between traditional healers and allopathic providers.

**Methods:**

We conducted 27 in-depth interviews and 133 surveys with a random sample of traditional healers living in Bushbuckridge, South Africa, where anti-retroviral therapy (ART) is publicly available, to learn: (1) healer perspectives about which HIV patients they choose to treat; (2) the type of treatment offered; (3) outcomes expected, and; (4) the cost of delivering treatment.

**Results:**

Healers were mostly female (77%), older (median: 58.0 years; interquartile range [IQR]: 50–67), with low levels of formal education (median: 3.7 years; IQR: 3.2–4.2). Thirty-nine healers (30%) reported being able to cure HIV in an adult patients whose (CD4) count was >350cells/mm^3^. If an HIV-infected patient preferred traditional treatment, healers differentiated two categories of known HIV-infected patients, CD4+ cell counts <350 or ≥350 cells/mm^3^. Patients with low CD4 counts were routinely referred back to the health facility. Healers who reported offering/performing a traditional cure for HIV had practiced for less time (mean = 16.9 vs. 22.8 years; *p* = 0.03), treated more patients (mean 8.7 vs. 4.8 per month; *p* = 0.03), and had lower levels of education (mean = 2.8 vs. 4.1 years; *p* = 0.017) when compared to healers who reported not treating HIV-infected patients. Healers charged a median of 92 USD to treat patients with HIV.

**Conclusion:**

Traditional healers referred suspected HIV-infected patients to standard allopathic care, yet continued to treat HIV-infected patients with higher CD4 counts. A greater emphasis on patient education and healer engagement is warranted.

## Background

In 2015, there were 19 million people living with HIV in eastern and southern Africa, including nearly 1 million people newly infected by the disease [1]. In rural sub-Saharan Africa, people living with HIV frequently seek allopathic clinical services while they concurrently receive treatment from traditional healers [2–6]. In the rural Bushbuckridge sub-district of Mpumalanga Province, South Africa, 69% of HIV infected patients report routinely visiting both types of practitioners to treat their HIV disease, opportunistic infections (Tuberculosis, Kaposi’s sarcoma), and side effects from antiretroviral medication (ART) [2]. As knowledge about HIV transmission and allopathic treatment practices becomes more widely understood, healers may be incorporating clinical information about CD4 cell counts, virial load, or the development of drug resistance into their own diagnostic and treatment strategies.

Traditional healers in sub-Saharan Africa are thought to be able to diagnose and treat physical and emotional ailments resulting from sources such as social transgressions, spirits, curses, and sorcery, that allopathic health care workers are unable to address [7–10]. Despite allopathic practitioner’s concerns with traditional medicine use, patients still seek, and often prefer, alternative treatments. Healers generally speak the same local languages as patients, spend more time with patients explaining the source of the illness and the necessary treatments, offer greater perceived compassion than clinicians, and are oftentimes more easily accessible [11]. When experiencing illness, it is common for patients to go back and forth between the allopathic health system and the traditional health system [2, 11–13]. Medical pluralism is common among patients dealing with a variety of chronic and acute ailments, including HIV, leading to poorer health outcomes among individuals who delay or abandon allopathic treatment for biomedical conditions [10, 14–18]. Although there is no legal framework to control healer treatment of HIV, with those claiming the ability to cure the disease censured by provincial or district health offices and by their own healer organizations.

Little work has been done to document HIV-related treatment provided by the traditional health system currently functioning within the rural Bushbuckridge area where the MRC/Wits Agincourt Research Unit has been running a Health and socio-Demographic Surveillance System (HDSS) since 1992. While we know some people employ traditional services for their HIV care and treatment, we know little of healer perspectives about which HIV patients they choose to treat, the type of treatment offered, outcome expected, and the cost of treatment. This study was undertaken to address this paucity.

## Methods

### Study location

The study was conducted in the Agincourt sub-district, Mpumalanga province, northeastern South Africa. The MRC/Wits Agincourt Research Unit oversees the maintenance and operation of the Agincourt HDSS. Roughly 500 km northeast of Johannesburg, the unit has been engaged in population-based health and demographic research since 1992 [19–21]. The Agincourt HDSS population is comprised of roughly 115,000 individuals –mainly xiTsonga-speaking– spread throughout 21,000 households in 31 research villages. Roughly one-third of the permanent HDSS population is comprised of former Mozambican refugees, who immigrated to South Africa during the 1980s. The study area contains eight government run clinics, a public-private community health center, and one large public health center. Patients are referred to three district hospitals, each located at 25–55 km from the site and relying primarily on public means of transport. HIV testing and treatment is available at all public health care facilities in the Agincourt sub-district. Therapy initially became available at district hospitals in 2007, with a complete roll-out of ART to satellite clinics by 2010.

### Study population

Traditional healers who are registered with Kukula organization [22] were randomly selected using the random selection function in Stata® (StataCorp, LP, College Station, Texas, USA). The Kukula organization consists of more than 300 traditional healers who have organized to champion for healer rights and responsibilities in the Bushbuckridge region. The majority of healers living in the area are members. Of the 169 participants (from a population of 280) selected to participate, four were no longer living in the area and five had died. Of the 160 healers who were located and approached to participate in the study, all accepted (100% acceptance rate).

### Ethics, consent and permissions

This study was approved by the Vanderbilt Institutional Review Board (IRB # 140646), the University of Witwatersrand Institutional Review Board (IRB #140547), and the Mpumalanga Department of Health’s Research Ethics Committee. All participants provided written informed consent.

### Data collection

We conducted 27 in-depth interviews followed by 133 quantitative surveys between July 2014 and August 2015. We used the in-depth interviews to inform and further refine the development of the questionnaire used for the survey. Open, semi-structured interviews were conducted in xiTsonga by a trained qualitative field supervisor (SN; the other co-authors participated in several of the interviews) and were focused on generating data on how healers diagnosed illness, how they learned to treat HIV, how they decided which HIV-infected patients to accept for treatment, and whether they believed they could cure the HIV virus from infected patients. Traditional healers were also asked about the ability of allopathic medicine to treat HIV. Interviews lasted an average of 61 min. Quantitative surveys, borne from the findings of our qualitative interviews, asked healers how they diagnosed HIV, preferred treatment strategies, anticipated outcomes, and costs charged to patients. Data was also collected on the number and type of patient diagnoses in the past seven and 30 days. Given limitations in healer literacy, survey data was collected via face-to-face interviews with healers at their preferred location.

### Data analysis

Survey participant characteristics and service uptake were presented as frequencies with percentages or medians with interquartile ranges (IQR). Multivariable log-binomial regression was used to model the odds of treating HIV. The model was adjusted for age and education level.

Qualitative data were transcribed within two weeks of the interview into xiTsonga and were subsequently translated into English. Two researchers participated in the thematic analysis, both with extensive experience conducting qualitative research. Agreement in coding was reviewed and discrepancies resolved by consensus. A comparison of coding agreement on the first six interviews found 90% agreement using Cohen’s Kappa in MAXQDA® (VERBI GmbH, Berlin, Germany) software.

## Results

### Traditional healer demographics

Among healers who participated in the quantitative surveys, healers in the rural Bushbuckridge were mostly female (77%), older (58 years [SD: 14]), had been practicing traditional medicine for an average of21 years (SD: 14)), and had low levels of education (mean3.7 years [SD: 3]). Ninety-two healers (69%) self-identified primarily as an Inyanga (herbalist), with the remaining healers identifying as a Sangoma [divine treatment; 34 (26%)], herbalist [2 (1%)], or “other” [5 (4%)]. Among all healers, the mean number of patients treated in the past month was 6; however, among active healers (those seeing a least one patient per month), an average of 12 patients. The majority of healers did not identify with any particular religion (*n* = 68, 52%), but the most common religious affiliations were Zion (*n* = 19, 15%), Apostolic (*n* = 11, 9%), and Bandla Lama Nazaretha (*n* = 10, 8%). Immigration from Mozambique (either themselves or their parents) was reported by 94 (71%) of healers; however, only 4 (3%) reported speaking fluent Portuguese. The most commonly reported languages spoken included: xi-Tsonga (*n* = 130, 98%), isiZulu (*n* = 40, 30%), English (*n* = 12, 9%), and Xhosa (*n* = 8, 6%).

### Diagnosis and treatment

When initiating the diagnosis process, healers described the legal rationale of accompanying the patient to the allopathic health care facility for HIV and TB testing. “*Yes, it is a law that if a patient comes to us we have to refer them to the hospital to be tested. We are not allowed to treat a patient without being tested ...*” (female, unknown age). Despite the health regulation requiring them to refer all patients suspected of having HIV to clinical sites for HIV counseling and testing, healers acknowledged that they might unknowingly treat HIV if a patient lies about their status or refuses to test. As such, traditional healers often offer treatment for symptoms suggestive of HIV infection, without knowing whether or not a patient is HIV-positive. One healer noted, “*The problem with HIV is that you don’t know if you are treating it or not; because when a patient has diarrhea we give her treatment, when she is coughing or she had sores or anything, we just give her treatment to take. Then we end up not knowing if we are treating it [HIV] or not*” (female, 54 years).

Healers who claimed to cure HIV reported diagnosing the illness through traditional practices (throwing the bones, for example), while others discussed accompanying suspected HIV cases to the clinic for testing. Healers who brought patients to the health facility waited with them, and, if they tested HIV positive, ascertained their CD4 cell count from the patient. Qualitative interviews revealed healers differentiating between two categories of known HIV-infected patients: (1) those with CD4 < 350 cells/mm^3^ and (2) those with >350 cells/mm^3^. Healers were less likely to treat patients with a low CD4 for fear that they will die in their care: “*If the person is not bedridden I do treat her but when she comes to me being critical ill, I will not cure her because her CD4 count will be very low and they [her immune system] are failing to fight the virus*” (female, 50 years). Among those surveyed, only 18 (14%) reported successfully curing HIV-infected adult patients with CD4 counts <350 cells/mm^3^, while 39 (30%) reported successfully treating HIV-infected adult patients with CD4 cell >350 cells/mm^3^. Treatment strategies included providing herbal remedies to be ingested and/or injected. One healer describes the treatment process: “*I will give him two liters of the treatment [containing a mixture of six herbs]. He will take it and after two or three days he will start to have diarrhea and when he tells me that he has started to have diarrhea I will give him a due date; and I will ask him if they have the tree that I want him to dig its roots and when he says they have it I will tell him to dig or to eat its leaves… his diarrhea will stop and I will also tell him to stop taking the medication that I have given him. He has to come back and collect another treatment that will cure him. All the powers of things that will create AIDS will be gone after taking the treatment and having diarrhea.” (Male, 78 years).*

Healers who reported treating HIV-infected patients with >350 cells/mm^3^ (versus those who did not) treated more patients (8.7 vs. 4.8 per month; *p* = 0.03), had been practicing for less time (16.9 vs. 22.8 years; *p* = 0.03), and had lower levels of education (2.8 vs 4.1 years; *p* = 0.017) (Table 1). Inyangas had higher odds of treating someone with HIV versus Sangomas (OR: 2.49), but this was not statistically significant (*p* = 0.068). Modified Poisson regression using robust errors revealed Inyangas were more likely to treat (RR: 2.20 [CI: 1.00–4.83] *p* = 0.049) (Table 2). Being above the median age and increased educational attainment reduced the risk of treating HIV-infected patients but fell short of the 0.05 threshold for significance. Qualitative interviews revealed that leaves and roots from the Ximuwana, Gashu, and Nkompa trees were most commonly used to treat HIV symptoms. Healers charged a median of 92 USD (IQR: 43–123) to treat HIV patients with high CD4.

**Table 1:** Healer characteristics by claim to cure HIV

	Claims to cure	Cannot cure	Combined	*p*-value
Gender (female)	29 (28%)	74 (72%)	94 (71%)	0.584
Type of Healer				0.418
Sangoma	6 (15%)	28 (30%)	34 (26%)	
Inyanga	32 (82%)	60 (64%)	92 (69%)	
Herbalist	1 (3%)	1 (1%)	2 (2%)	
Other	0 (0%)	5 (5%)	5 (4%)	
Length of time practicing	16.9 (11)	22.8 (15)	21.1 (14)	0.031^a^
Education Attainment	2.8 (3)	4.1 (3)	3.7 (3)	0.017^a^
Family from Mozambique	30 (77%)	64 (69%)	94 (71%)	0.348
Age (Mean, SD)	55 (10)	60 (15)	58 (14)	0.084
Number of patients past month (mean, SD)	8.7 (9.9)	4.8 (9.0)	5.9 (9)	0.031^a^

^a^statistically significant

**Table 2:** Risk of intentionally treating HIV

	Risk ratio	Robust standard errors	95% CI	*p*-value
Type of Healer (Inyanga)	2.201	0.883	1.003–4.831	0.049^a^
Sex (Female)	0.791	0.238	0.439–1.427	0.436
Age Category (>median age)	0.617	0.183	0.346–1.102	0.103
Number of patients (per additional patient)	1.009	0.022	0.966–1.053	0.693
Education (per additional year)	0.904	0.134	0.812–1.006	0.006^a^

^a^statistically significant

### Referral to and from allopathic care providers

Healers were generally confident in their ability to treat certain conditions, but they also frequently referred patients to the health facility for HIV and TB testing, to treat dehydration/blood loss, and to assist patients they judged themselves as unable to cure. Among the healers in this study, 113 (85%) had referred a patient for dehydration or for a blood transfusion and 47 (36%) referred a patient to a clinic because they could not successfully treat them. While these transfers were common, they also led to patients shuttling back-and-forth between the two systems. One healer explains, “*When looking at the patient, I can see that this one doesn’t have water in his body because he will be very weak… What I do is to tell him to go to the hospital and get tested and after testing and have the results you will come back to me for treatment*” (Male, 67 years).

## Discussion

Traditional healers in Bushbuckridge, where ART is available at public hospitals and clinics, continue to treat HIV-infected patients for both HIV and other opportunistic infections resulting from HIV. Inyangas, or herbalists, were at higher risk of believing they could cure an HIV-infected patient than a Sangoma, an unexpected finding given the history of Sangomas treating HIV in South Africa [23]. Our data revealed the increasing sophistication of healer practices: healers selected which HIV-infected patients to treat based on CD4 counts. Healers were avoiding the treatment of any perceived end-stage terminal conditions, as the death of a patient is bad for business. Furthermore, treating HIV-infected individuals seemingly resulted in increased business. Healers claimed that they could cure HIV, leaving patients with no negative health implications after treatment, a potentially more tempting offer to patients than ART for life. Engagement with healers may need to focus on healer’s ability to both appreciate and subsequently advertise their own survival rates to gain entry into the traditional system.

Traditional healers were universally hesitant to disclose their strategies and sources of medication to treat, highlighted their concern with the theft of their intellectual property [24]. They discussed the use of unspecified herbs (ingested and injected), baths, and ceremony to cure the patient from HIV. The use of razors to inject herbs under the skin of patients is concerning, given the risk of HIV transmission from the patient to the healer [25].

The cost of traditional treatment is high, considering both median income and that primary health care, including antiretroviral therapy, [21] is free to the patient in South Africa. A recent study from South Africa exploring the cost of traditional healer treatment among people with epilepsy found costs to be somewhat lower than HIV treatment, but still substantially higher than allopathic health care costs [26]. While we cannot identify the number or proportion of HIV-infected patients paying these high fees, qualitative interviews and previous research in Bushbuckridge suggest a large number of patients do accept their treatment [2].

Traditional healers do refer patients to the allopathic health care system to ensure that patients suspected of being HIV-infected are tested and, in some cases, treated for HIV. In this study, at least 85% of healers had previously referred patients to allopathic services. This interaction highlights, at least, some current level of engagement between the two systems and while this study suggests that this engagement is possibly a result of fear of legal prosecution, further research on whether voluntary engagement is warranted given the strong relationship between community members and traditional healers, and the large number of registered healers in the Bushbuckridge area. Further engagement could facilitate the incorporation of their work into the allopathic health system. If trained and effectively engaged, traditional healers could help increase early diagnosis and therapy uptake with prompt referrals, adherence support, and avoidance of herb-drug interactions and toxicity, [6, 9, 10, 27–33] ultimately improving health outcomes for people with HIV in an region beset with extremely high levels of HIV [19].

Efforts to increase healer knowledge about specific diseases, improve relationships with allopathic practitioners, and reduce delays to allopathic care have been piloted with traditional healers in diverse countries, including Brazil, Uganda, Kenya, Ghana, Cameroon, Lesotho, Gambia, Nepal, and India [33–48] with mixed results.

Our random sample of traditional healers living in northeastern rural South Africa allowed us to generate data that reflects the source population. However, several factors may limit our results. Recall bias may constrain a healer’s ability to remember the number of patients (and their diagnosis) treated in the past 30 days. Social desirability bias may have resulted in fewer healers reporting the treatment of HIV given healer’s knowledge that treating HIV through traditional means is viewed negatively in South Africa. The interviewer attempted to reduce this bias by assuring healers that the data would not be reported to local authorities nor was she associated with any of the local health facilities.

## Conclusion

Traditional healers in rural South Africa are continuing to treat HIV symptoms in patients, often at prices much higher than the free primary allopathic healthcare available in the area. Further understanding of commonalities and differences between traditional and allopathic health care systems, ways of ensuring risk reduction among the traditional healers, and promotion of honest and targeted dialogue between the two systems may allow for greater coordination between the two systems and an ultimate improvement in HIV patient care in rural South Africa.

## Abbreviations

ART: Anti-retroviral therapy
CD4 cells: Cluster of differentiation 4
HDSS: Health and socio-Demographic Surveillance System
HIV: Human immunodeficiency virus
IQR: Interquartile range
